# Differentiated Effects of Robot Hand Training With and Without Neural Guidance on Neuroplasticity Patterns in Chronic Stroke

**DOI:** 10.3389/fneur.2018.00810

**Published:** 2018-10-08

**Authors:** Xin Wang, Wan-wa Wong, Rui Sun, Winnie Chiu-wing Chu, Kai-Yu Tong

**Affiliations:** ^1^Department of Biomedical Engineering, The Chinese University of Hong Kong, Shatin, Hong Kong; ^2^Department of Imaging and Interventional Radiology, The Chinese University of Hong Kong, Shatin, Hong Kong; ^3^Brain and Mind Institute, The Chinese University of Hong Kong, Shatin, Hong Kong

**Keywords:** long-term training effect, motor imagery, action observation, motor recovery, EEG discriminant rate, resting state fMRI, temporal variability, brain network

## Abstract

Robot-assisted training combined with neural guided strategy has been increasingly applied to stroke rehabilitation. However, the induced neuroplasticity is seldom characterized. It is still uncertain whether this kind of guidance could enhance the long-term training effect for stroke motor recovery. This study was conducted to explore the clinical improvement and the neurological changes after 20-session guided or non-guided robot hand training using two measures: changes in brain discriminant ability between motor-imagery and resting states revealed from electroencephalography (EEG) signals and changes in brain network variability revealed from resting-state functional magnetic resonance imaging (fMRI) data in 24 chronic stroke subjects. The subjects were randomly assigned to receive either combined action observation (AO) with EEG-guided robot-hand training (Robot_EEG_AO_, *n* = 13) or robot-hand training without AO and EEG guidance (Robot_non−EEG_Text_, *n* = 11). The robot hand in Robot_EEG_AO_ group was activated only when significant mu suppression (8–12 Hz) was detected from subjects' EEG signals in ipsilesional hemisphere, while the robot hand in Robot_non−EEG_Text_ group was randomly activated regardless of their EEG signals. Paretic upper-limb motor functions were evaluated at three time-points: before, immediately after and 6 months after the interventions. Only Robot_EEG_AO_ group showed a long-term significant improvement in their upper-limb motor functions while no significant and long-lasting training effect on the paretic motor functions was shown in Robot_non−EEG_Text_ group. Significant neuroplasticity changes were only observed in Robot_EEG_AO_ group as well. The brain discriminant ability based on the ipsilesional EEG signals significantly improved after intervention. For brain network variability, the whole brain was first divided into six functional subnetworks, and significant increase in the temporal variability was found in four out of the six subnetworks, including sensory-motor areas, attention network, auditory network, and default mode network after intervention. Our results revealed the differences in the long-term training effect and the neuroplasticity changes following the two interventional strategies: with and without neural guidance. The findings might imply that sustainable motor function improvement could be achieved through proper neural guidance, which might provide insights into strategies for effective stroke rehabilitation. Furthermore, neuroplasticity could be promoted more profoundly by the intervention with proper neurofeedback, and might be shaped in relation to better motor skill acquisition.

## Introduction

Stroke-induced disabilities often imperil the independence of stroke survivors and increase burden of care on their caregivers. Effective stroke rehabilitation is therefore in great demand to help stroke survivors to regain their independence and to relieve the burden. Robot-assisted therapy is now emerging and has been proved to exhibit encouraging effects on upper-limb motor recovery (1–4), by utilizing robotic device to facilitate reiterative movement training with high precision and intensity. Moreover, there is growing evidence suggesting that clinical recovery is attributed to neuroplastic reorganization, and the neuroplasticity can be promoted by specific interventions (5–7). A number of interventions, such as robot-hand training combined with neurofeedback system, have been adopted to facilitate neuroplastic changes and enhance recovery potential. This kind of approach usually asks subjects to perform motor imagery, detects their movement intentions from real-time electroencephalography (EEG) signals, and a robotic device is triggered when desirable EEG features are detected. Such neurofeedback system has been suggested to possess the capability of inducing neural plasticity (8). However, the recovery following this strategy can still be various across the stroke patients (2, 9). One of the challenges is that the interpretation of motor imagery can be varied from person to person, leading to diverse treatment outcomes. Action observation (AO) could be a possible means to overcome this challenge, which has been shown to have potential of rebuilding motor function by involving similar brain regions to motor execution. The impaired motor system after stroke could become accessible by recruiting the shared motor circuits during AO. The motor training effects on post-stroke motor memory formation could also be further augmented by observing an action congruent to the practiced task during physical training in order to facilitate the brain to have better motor relearning. Furthermore, from EEG studies, AO is found to be associated with mu suppression which the brainwave at around 10 Hz over the sensorimotor areas is suppressed significantly while observing motor acts performed by the others (10, 11). Therefore, an interactive brain-computer platform was built based on real-time detection of significant mu suppression from EEG signals. Neural guided training was provided to the subjects to train them using the right signals from the ipsilesional motor areas to control the system and activate the robot hand. The training effects on paretic upper-limb motor function were evaluated by Fugl-Meyer Assessment for upper-extremity (FMA-UE) which was repeatedly assessed before, immediately after and 6 months after the intervention, and were compared to a robot-hand training without AO and the input of the subjects' EEG signals.

Apart from evaluating behavioral changes using clinical assessments, more understanding of the neural mechanisms that underpins a restorative approach to stroke rehabilitation is also important. It is because the cerebral cortex plays a critical role in human locomotor functions. Here, EEG and functional magnetic resonance imaging (fMRI) were used to study the post-stroke neuroplasticity mechanisms after the interventions. In this study, EEG is not only used to detect the voluntary motor intention for brain-computer interface, but also used to explore the brain discriminant ability between motor imagery state and resting state during the early training stage and late training stage of the intervention. Brain discriminant ability can be referred as an index of discriminating brain activity between different mental states. Higher value of discriminant index represents a more prominent mental state transformation with evident brain activity features across different mental states, while lower value indicates no distinguishable features that can characterize the mental states. Stroke subjects might have difficulty in switching between the resting state and the task state like motor imagery. Hence this index has been used to detect motor imagery or to indicate motor imagery performance in brain-computer interface training for stroke recovery (2, 12). Classification techniques such as linear discriminant analysis (LDA) and support vector machines have been adopted to identify changing topographies during natural motor behaviors (13), brain state dynamics during neurodevelopment (14) and brain features during brain-computer interface control (15). An increase in classification accuracy under the same classification model after intervention may indicate an improvement in brain state transformation ability, demonstrating that different mental states can be classified more clearly and separately after training with the same classification model used. This brain state transformation ability, also named brain discriminant ability, may provide us more insights into the brain reorganization after the interventions.

Besides EEG, fMRI which employs blood-oxygen-level dependent (BOLD) contrast can provide high-spatial-resolution details of neuroplasticity mechanisms during neural reorganization (16). Resting state functional connectivity derived from fMRI data has been widely used to explore the brain activity in the absence of an explicit task, which subject stays still without any external stimuli or tasks (17). Conventional analysis methods usually use the whole period of resting state fMRI data and compute the average functional connectivity representing the resting brain activity. However, brain activity fluctuates even during resting state and the spontaneous fluctuations have been reported to be related to vigilance (18), arousal (19), and consciousness (20). Recent studies tried to use dynamic functional connectivity to characterize various neurological diseases (21–23). The signs of showing brain dynamics from the spontaneous fluctuations of the resting brain activity (24–26) might be related to motor relearning and brain adaptation during stroke recovery process. Therefore, dynamic functional connectivity analysis could be superior to the static way to look at the resting brain activity which the brain-network functions can fluctuate on a time scale from seconds to minutes. Thus, studying temporal variability of resting brain activity may provide new information of brain-network interactions during the reorganization.

The temporal changes of brain activity can be characterized as flexibility or variability. A network flexibility derived from multilayer approach was used to reveal network reconfiguration during linguistic processing (27). Other study used temporal variability of brain networks to reveal the differences between healthy brains and brains with mental disorders (28), and found out that the variability can be a biomarker to distinguish between patients and healthy subjects. In fact, the term variability has been widely used in different brain researches, such as applying internetwork correlation to study functional variability from childhood to adulthood (29), and studying modular variability in relation to cognitive flexibility (30). These studies uncovered that the brain regions with different variability changes may be involved in different modes of information transformation under different situations.

In this study, two neuroimaging modalities, including EEG and fMRI, were employed to evaluate the neuroplasticity changes in chronic stroke subjects after interventions using two different kinds of motor imagery robot hand training paradigm: with AO plus real-time EEG guidance and without AO and EEG guidance. Motor function of the paretic upper-limb was evaluated at three time-points: before, immediately after and 6 months after the interventions. The brain discriminant ability between motor imagery state and resting state revealed from EEG data, and the brain network variability revealed from resting-state fMRI data were studied and used to indicate the neuroplasticity changes. We hypothesized that the stroke subjects with neural guided training would have a sustainable improvement in motor functional recovery and a significant neuroplasticity change in the brain discriminant ability and the brain network variability after the intervention compared with the subjects without guided training. Such findings may provide us a better understanding of neural mechanisms during stroke recovery from the two different training strategies.

## Materials and methods

### Participants

Twenty-four chronic stroke subjects (20 males and 4 females; mean age = 54 ± 9 years) were recruited from local community. All of them suffered from first-ever stroke. The inclusion criteria were: (1) sufficient cognition to follow experimental instructions with Mini–Mental State Examination (MMSE) score >21, (2) moderate to severe motor impairments at the paretic upper limb (Fugl-Meyer Assessment score for upper-extremity less than 47) (31), and (3) hemiparesis resulting from a single unilateral brain lesion with stroke onset more than 6 months before data collection. Exclusion criteria were: (1) severe hand spasticity (the spasticity during extension of the finger joints was more than 3 as assessed by Modified Ashworth Scale) (1), open hand wound or hand deformity, (2) visual field deficits, (3) aphasia, neglect, and apraxia, (4) participation in any therapeutic treatment (“outside therapy”) performed with the affected upper limb during the course of the study, (5) history of alcohol, drug abuse, or epilepsy, and (6) bilateral infracts, uncontrolled medical problems, and serious cognitive deficits.

All subjects completed a 20-session robot hand training with simultaneous EEG signal recording. However, only 16 out of 24 subjects who had no MRI contraindications were able to complete MRI scan. Motor functions of paretic upper limb of stroke subjects were assessed at three time-points (before, immediately after and 6 months after intervention) by trained clinical assessors who were blinded to the experiment. Fugl-Meyer Assessment for upper-extremity (FMA-UE) (32) was used to evaluate and measure the upper limb motor function. The changes in the FMA-UE were compared against minimal clinically important difference (MCID) (33) which was set at 4 based on sensitivity analysis (34). The MCID is defined as the smallest change in a treatment outcome that would be identified as important by a patient, and provides a threshold above which the patient would experience the outcome as relevant. The study was approved by the Joint Chinese University of Hong Kong-New Territories East Cluster Clinical Research Ethics Committee. Each subject gave informed consent before the experiment.

### Interventional protocols

All subjects received a 20-session robot-assisted hand training, with an intensity of 3–5 sessions per week that was completed within 5–7 weeks. During each session, 100 repetitive hand movements were performed by each subject with intermittent rest after every 10 trials. A robot hand providing mechanical support was used to assist the subject in completing hand grasp/open task during the training (1). Subjects were randomly assigned to one of the two groups: (1) Robot_EEG_AO_ Group: Action observation and motor imagery during playback of video of biological movement with real-time EEG guidance to trigger the robot hand. The subjects were asked to observe a video demonstrating either grasping or releasing a cup using the subjects' unaffected hand, and the video frames were flipped to pretend that the subjects were observing their affected hand to do those hand actions. Robot hand was triggered to help hand open or grasp if mu suppression calculated from real-time EEG signals was above 20 (35). The calculation of mu suppression can be found in EEG preprocessing part. (2) Robot_non−EEG_Text_ Group (sham group): Motor imagery during display of text instruction of movement without EEG guidance and the robot hand was triggered randomly. The subjects were instructed to imagine their affected hand movements during a text cue of showing “hand open” or “hand grasp”. Robot hand was randomly triggered regardless of the subjects' EEG signals. In order to maintain the two groups having comparable training intensity for the paretic hand, the “success rate” of triggering the robot hand in Robot_non−EEG_Text_ Group was set as 80%, which was the similar level as that in Robot_EEG_AO_ Group from our preliminary study. All subjects were instructed to imagine the same movement with the affected hand during the video or text display. The display of experimental sequences for the two training paradigms was controlled by the Psychophysics Toolbox 3.0 (http://psychtoolbox.org/). The experimental paradigm was shown in Supplementary Figure 1.

### EEG and MRI data acquisition

The EEG signals were captured continuously and simultaneously by an amplifier (g.USBamp, g.Tec Medical Engineering GmbH, Austria) with 16 active electrodes (g.LADYbird, g.Tec Medical Engineering GmbH, Austria) during the robot-assisted hand training in both groups. Sixteen electrodes were placed over the motor related regions at the central area according to the international 10–20 system (C1, C2, C3, C4, C5, C6, Cz, FC1, FC2, FC3, FC4, FCz, CP1, CP2, CP3, CP4). EEG signals were referenced to a unilateral earlobe, grounded at a frontal position (Fpz), with a sampling rate of 256 Hz, and a band-pass filtering (2–60 Hz) and a notch filtering (48–52 Hz) applied. Transmission impedance was kept below 1 kOhm with conductive gel for all electrodes.

Sixteen subjects who had no MRI contraindications had MRI scans before and after the intervention with eight subjects in each group. A 3T Philips MR scanner (Achieva TX, Philips Medical System, Best, Netherlands) with an 8-channel head coil was used to acquire high resolution T1-weighted anatomical images (TR/TE = 7.47/3.45 ms, flip angle = 8°, 308 slices, voxel size = 0.6 × 1.042 × 1.042 mm^3^) using a T1-TFE sequence (ultrafast spoiled gradient echo pulse sequence), and BOLD fMRI images (TR/TE = 2,000/30 ms, flip angle = 70°, 37 slices/volume, voxel size = 2.8 × 2.8 × 3.5 mm^3^) using a FE-EPI sequence (gradient-echo echo-planar-imaging sequence). Subjects were presented with a white crosshair in black background and instructed to rest while focusing on the fixation cross during the fMRI acquisition. The resting state fMRI acquisition lasted for 8 min.

### EEG data preprocessing and analysis

#### Online data analysis

Only for the subjects who were presented with video display (Robot_EEG_AO_ group), their EEG signals were processed in real time to provide EEG guidance to the subjects. The mu rhythm is mainly found over the vertex (EEG Cz electrode location) or laterally across the precentral motor cortex, normally at C3 or C4 electrode depending on which hand or arm movement is being performed or visualized contralaterally (36). The mu rhythm occurs when a person is at rest, and it is suppressed when the person executes a motor action or views a motor action performed by the other. Since it has been suggested that restitution of near-normal circuitry might be the best basis for better functional recovery for stroke survivors (37–39), either C3 or C4 electrode was selected to compute the mu suppression according to the subjects' brain lesion side for promoting their motor relearning patterns similar to the normal patterns. The mu suppression is believed to be associated with the activation of mirror neuron system and has been used in motor imagery study (40). Hence it can be used as an indicator of mirror neuron activity to provide neurofeedback to the participant. Fast Fourier transform with a Hanning window covering the EEG data during the video display (6 s) was applied to convert the EEG signals to the frequency domain. The mean power in the mu band (8–13 Hz) of the EEG signal was calculated to get the mu suppression score. Then the mu suppression score was calculated using the following Equation (41):

$$\begin{array}{l} \text{MSS}=\;-\frac{{\text{P}}_{\text{AO}}-{\text{P}}_{\text{rest}}}{{\text{P}}_{\text{rest}}}\times 100 \end{array}$$

where MSS represents the mu suppression score, P_AO_ and P_rest_ represent the mu power of EEG signals during AO and rest, respectively. Robot hand was triggered to help hand grasp or open if the mu suppression score is >20, which means that the ratio of the mu power between observation and rest was below 80% according to the average results of healthy subjects (35).

#### Offline data analysis

**Figure 1:**
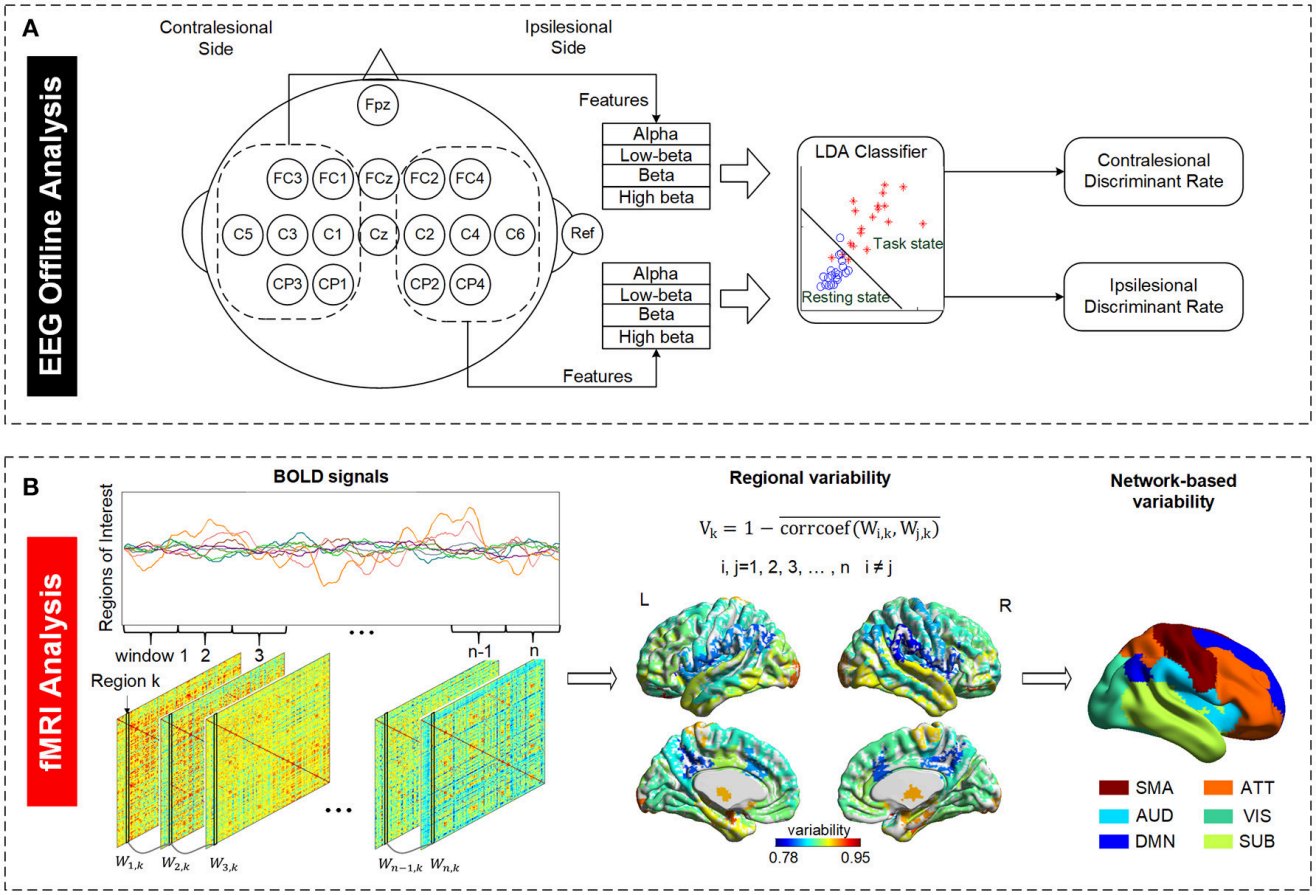
Illustration of EEG and fMRI data analysis. **(A)** Definition of EEG discriminant rate based on the ipsilesional and contralesional EEG signals. The ipsilesional and contralesional discriminant rates were calculated based on the ipsilesional and contralesional EEG signals, respectively, during motor imagery state (task state) relative to the respective EEG signals during resting state. The subject with right brain lesion was used as an example in the figure. **(B)** Definition of temporal variability derived from resting state fMRI data. A regional variability is defined as the variation in functional connectivity profiles of that region across different time windows. The variability indices of all regions were regrouped into six functional subnetworks for further examination. SMA, sensory-motor areas; ATT, attention network; AUD, auditory network; VIS, visual recognition network; DMN, default mode network; SUB, subcortical network.

For all subjects in both groups, EEG signals were analyzed offline to investigate motor imagery performance, which is represented as an ability of discriminating between motor imagery state and resting state. We defined a discriminate rate (DR) to reflect this ability. LDA classifier, a linear classifier which usually works well in EEG signal classification compared to other models such as principal component analysis (PCA), independent component analysis (ICA) or support vector machines (42, 43), was used to calculate DR in this study. It has been used in a number of brain-computer interface (BCI) studies such as motor imagery based BCI (44), P300 speller (45), or asynchronous BCI (46). The EEG data of the first four training sessions were used to calculate the DR to reflect the brain discriminant ability at early training stage while the last four training sessions were used to calculate the DR to reflect the brain discriminant ability at late training stage. We randomly chose two sessions of the EEG dataset from the first and last four sessions separately as the training set and the remaining two sessions in the respective four sessions as the testing set. The purpose of the training set was to estimate the subject dependent parameters of LDA classifier which are the coefficients of the classifier and distinct among subjects for characterizing their own brain activities. Based on classifier model built from the training set, motor imagery performance was estimated using the testing sets. As shown in Figure 1A, the EEG signals from electrodes in the contralesional hemisphere were used to calculate the contralesional-DR while the EEG signals from ipsilesional hemisphere were used to calculate the ipsilesional-DR. This is for studying whether there is an improvement in the motor imagery performance based on the ipsilesional and/or the contralesional EEG signals after the interventions. Alpha (8–13 Hz), Low-beta (12.5–16 Hz), Beta (16.5–20 Hz), and High-beta (20.5–28 Hz) powers of EEG signals averaged from all electrodes in the ipsilesional hemisphere or contralesional hemisphere were served as the four features input into LDA classifier model to calculate the ipsilesional-DR or contralesional-DR of each stroke subject, respectively. Features of all trials in each session were averaged to avoid bias.

### MRI data preprocessing and analysis

#### Functional MRI data preprocessing

The fMRI data were preprocessed and analyzed using Analysis of Functional NeuroImages (AFNI) software (http://afni.nimh.nih.gov/afni). The analysis steps followed the recommended analysis procedures for resting state fMRI data (47, 48). The first 10 volumes of each subject's fMRI data were removed to assure that the remaining volumes in the data were at magnetization steady state. Despiking of large transients, slice-timing correction and motion correction with six-parameter rigid body transformation were done for the remaining 230 functional brain volumes. Then the anatomical dataset was aligned to the functional dataset. Spatial normalization of the T1 images registered to MNI152 template in MNI space and a 4 mm full-width-at-half-maximum (FWHM) isotropic Gaussian kernel smoothing were applied. The time series from lateral ventricles and white matter were derived for nuisance regression by segmenting the T1 image into gray matter, white matter, and CSF using FreeSurfer for each subject (49). Other nuisances included motion parameters and motion parameter time derivatives. In the meantime, volumes with excessive motion were censored if the Euclidean norm of the derivatives of the motion parameters exceeded 0.2 by using the function *regress_censor_motion* in AFNI. Bandpass filtering (0.009–0.08 Hz) were also applied simultaneously. For group statistical analysis, subjects with left-hemispheric lesions were flipped along the midsagittal plane, so that the lesions of all subjects were in the right hemisphere.

#### Regions of interest definition

The whole-brain temporal variability changes were studied in chronic stroke patients before and after the interventions. It is common to apply popular standard brain atlas, such as automated anatomical labeling (AAL) atlas or Harvard-Oxford (HO) atlas to perform brain segmentation. However, varied lesion extent and multiple lesion areas of stroke subjects could render such standard atlas less accurate. Therefore, for the T1-weighted anatomical image, white and gray matter segmentation was done for each subject using FreeSurfer (Athinoula A. Martinos Center for Biomedical Imaging, USA) to partition the brain volume to generate outputs with labels corresponding to the white matter, the cortex, and the deep gray nuclei (17). After the brain segmentation, the parcellated regions constructed by FreeSurfer were remapped to AAL template, a total of 84 regions overlapping with the AAL template were chosen, including cortical and subcortical areas. Six functional subnetworks, including default mode network, attention network, visual recognition network, auditory network, sensory-motor areas, and subcortical network, were used to regroup the 84 regions according to the classification method of Tao et al. (50) for studying the temporal variability of brain regions in the subnetworks with similar functions and dense functional connectivity with each other. Supplementary Table 1 lists all the regions of interest and their corresponding subnetworks.

#### Temporal variability analysis

The mean BOLD time series was extracted for each brain region, and all the BOLD signals of all 84 regions were partitioned into non-overlapping time windows. As illustrated in Figure 1B, an 84^*^84 adjacency matrix W was calculated for each window. Each element of W represents the Pearson correlation of two regions in that window. The temporal variability of a region of interest k is defined as follows (28):

$$\begin{array}{l} {\text{V}}_{\text{k}}=1-\bar{\text{corrcoef}\left({\text{W}}_{\text{i},\text{k}},\;{\text{W}}_{\text{j},\text{k}}\right)}\;,\text{ i},\text{ j}=1,\;2,\;3,\ldots ,\text{ n},\text{ i}\neq \text{j} \end{array}$$

where V_k_ stands for the variability of region k, i, and j refer to the i^th^ and the j^th^ window, respectively. Therefore, the variability of a region characterizes the mean correlation of temporal functional changes at different time windows. Here Pearson correlation coefficient was used as the measurement of functional connectivity.

To reduce the effect from randomly choosing window length, the variability values of a certain brain region across different window lengths (10, 12, 14, …, 26, 28, 30 time points, corresponding 20, 24, …, 52, 56, 60 s, TR = 2 s) were averaged for each subject. The reason of choosing this range of window lengths was that these window lengths can capture rapidly temporal characteristics and get rid of noise influence (24, 51–53). To test the consistency of different window lengths, we investigated the correlations between the variability values calculated with these window lengths, and found that the variability values were highly correlated (*r* > 0.97, Supplementary Figure 2). This indicated that variability values of chosen window lengths can be averaged together as suggested by Zhang et al. (28).

### Statistical analysis

The statistical tests were conducted using either SPSS 19 (IBM SPSS, NY, US) or Excel. Friedman test was used to evaluate the long-term changes in the FMA-UE scores before, immediately after and 6 months after the intervention for each group separately. Non-parametric Wilcoxon signed-rank test was used as *post-hoc* test to examine significant changes of different combinations of three time-points for FMA-UE scores. The Scheirer-Ray-Hare test which is a 2-way non-parametric analysis of variance (ANOVA) was applied to examine whether the FMA-UE score was influenced by time and group factors. Paired *t*-test was used to reveal any significant changes in the ipsilesional and contralesional EEG discriminant rate for each group separately. A two-way repeated-measures ANOVA with “time” (pre-training vs. post-training) and the temporal variability of brain regions within the same functional subnetworks as within-subject factors was used to assess the training effects. The Greenhouse-Geisser adjustment was applied to the degrees of freedom for all analyses if the Mauchly's test of sphericity was significant. A paired *t*-test was applied to each voxel in standard MNI space to find the regions with significant changes in variability before and after the intervention. A one-way multivariate analysis of variance (MANOVA) was used to test the statistical difference in the changes of fMRI temporal variability of six brain subnetworks between the two subject groups.

## Results

### Clinical characteristics

**Figure 2:**
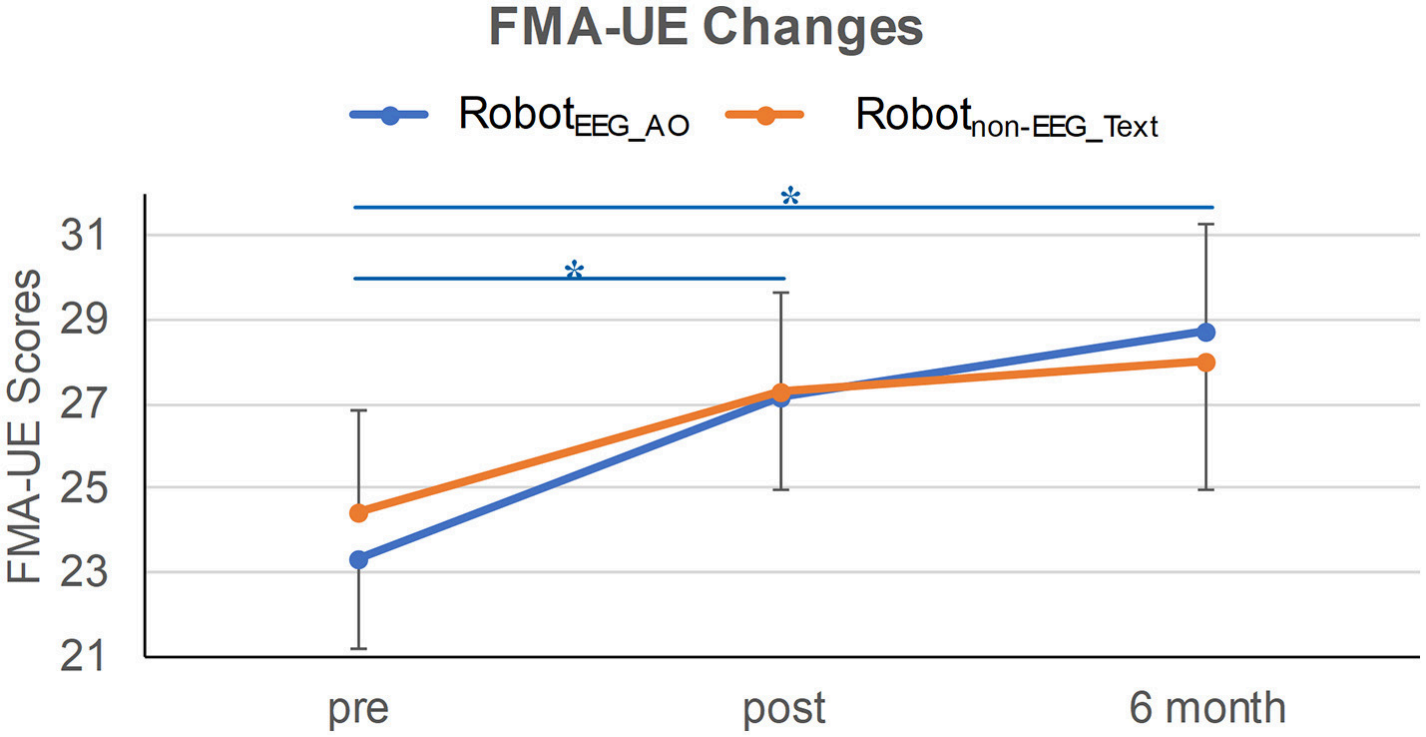
FMA-UE changes in the two training groups at three time-points. Significant improvement in the paretic motor functions was revealed between pre- and post-intervention, and between pre-intervention and 6-month follow-up in Robot_EEG_AO_ group. Error bar stands for the standard error. Asterisk (^*^) indicates that significant difference was observed at *p* < 0.05.

Demographics and clinical characteristics of the stroke participants are listed in Table 1. Before the interventions, there was no significant difference in the clinical score between the two groups (FMA-UE: *p* = 0.772). After the interventions, only Robot_EEG_AO_ group showed significant difference in paretic upper-limb motor functions across the longitudinal evaluation [χ^2^(2) = 7.659, *p* = 0.022] while no significant difference in the motor functions was revealed in Robot_non−EEG_Text_ group [χ^2^(2) = 4.537, *p* = 0.103]. *Post-hoc* analysis using Wilcoxon signed-rank tests showed that there were significant improvements in the paretic motor functions between pre- and post-intervention (*Z* = −2.135, *p* = 0.033) and between pre-intervention and 6-month follow-up (*Z* = −2.451, p = 0.014) in the Robot_EEG_AO_ group. However, no significant difference in the motor functions was found between post-intervention and 6-month follow-up (*Z* = 1.682, *p* = 0.092) for the same subjects. The proportion of stroke subjects exceeding the MCID (that was 4 for FMA-UE) was higher in the Robot_EEG_AO_ group (pre vs. post: 53.8%; pre vs. 6-month: 54.5%) compared with the Robot_non−EEG_Text_ group (pre vs. post: 36.4%; pre vs. 6-month: 36.4%). However, the effect of the interaction between factors time and group (*p* = 0.78), or the effect of group factor (*p* = 0.94), or the effect of time factor (*p* = 0.17) on the FMA-UE scores were all found insignificant from the results of the Scheirer-Ray-Hare test. The changes of FMA-UE scores for the two groups are illustrated in Figure 2.

**Table 1:** Demographics and clinical characteristics of the participants.

**Group**	**Subject**	**Age range**	**Gender**	**Lesion side**	**Lesion locations**	**Stroke Type**	**Stroke onset (*y*)**	**FMA-UE**		
								**Pre**	**Post**	**6 month**
Robot_EEG_AO_	S1	55–59	M	R	Brainstem	I	11	24	21	22
	S2	60–64	M	L	PLIC, putamen	I	11	22	24	24
	S3	45–49	M	R	MFG, SFG, precentral, supramarginal, SMA	I	1	19	34	28
	S4	65–69	M	L	Insula, putamen, IFG, temporal pole	H	8	22	27	32
	S5	65–69	M	R	Insula, ITG, IOG, putamen	H	1	13	16	27
	S6	45–49	M	R	ITG, MTG, STG, MOG, angular, supramarginal	H	0.67	17	25	25
	S7	60–64	M	R	Insula, putamen, rolandic operculum, IFG	I	3	16	14	18
	S8	50–54	M	L	MFG, precentral, IFG, postcentral, insula, SFG	I	1	41	36	40
	S9	45–49	F	R	Putamen, insula	I	1	36	41	48
	S10	45–49	M	L	NA	H	2	20	24	26
	S11	65–69	F	R	NA	I	2	25	26	26
	S12	65–69	M	R	NA	I	5	23	33	NA
	S13	30–34	M	R	Insula, STG, IFG, putamen, rolandic operculum, temporal pole	I	2	25	32	NA
Robot_non−EEG_Text_	S14	55–59	M	L	Insula, IFG, putamen	H	5	28	33	24
	S15	55–59	M	R	Insula, IFG, putamen, rolandic operculum, temporal pole	I	7	20	25	21
	S16	50–54	M	L	Putamen, caudate nucleus	I	1	24	22	22
	S17	40–44	M	R	Insula, rolandic operculum, IFG, STG, putamen, temporal pole	H	5	15	17	16
	S18	40–44	M	R	Insula, MTG, STG, putamen, temporal pole, rolandic operculum	H	3	17	20	20
	S19	55–59	M	R	Insula, rolandic operculum, IFG	I	6	13	23	20
	S20	50–54	F	L	Insula, rolandic operculum, putamen	H	3	34	34	37
	S21	45–49	M	R	Insula, putamen	H	1	34	37	35
	S22	55–59	M	L	NA	H	2	20	19	28
	S23	40–44	M	R	NA	I	2	33	31	50
	S24	55–59	F	L	NA	I	4	31	39	35
	Mean ± SD	54 ± 9					4 ± 3	24 ± 8	27 ± 8	28 ± 9

*y, year; M, male; F, female; R, right hemisphere lesion; L, left hemisphere lesion; IFG, Inferior frontal gyrus; IOG, Inferior occipital gyrus; ITG, Inferior temporal gyrus; MFG, Middle frontal gyrus; MOG, Middle occipital gyrus; MTG, Middle temporal gyrus; PLIC, Posterior limb of the internal capsule; SFG, Superior frontal gyrus; SMA, Supplementary motor area; STG, Superior temporal gyrus; H, hemorrhagic stroke; I, ischemic stroke; FMA- UE, Fugl-Meyer Assessment for upper-extremity (maximum: 66); SD, standard deviation; NA, not available*.

### EEG discriminant rate

**Figure 3:**
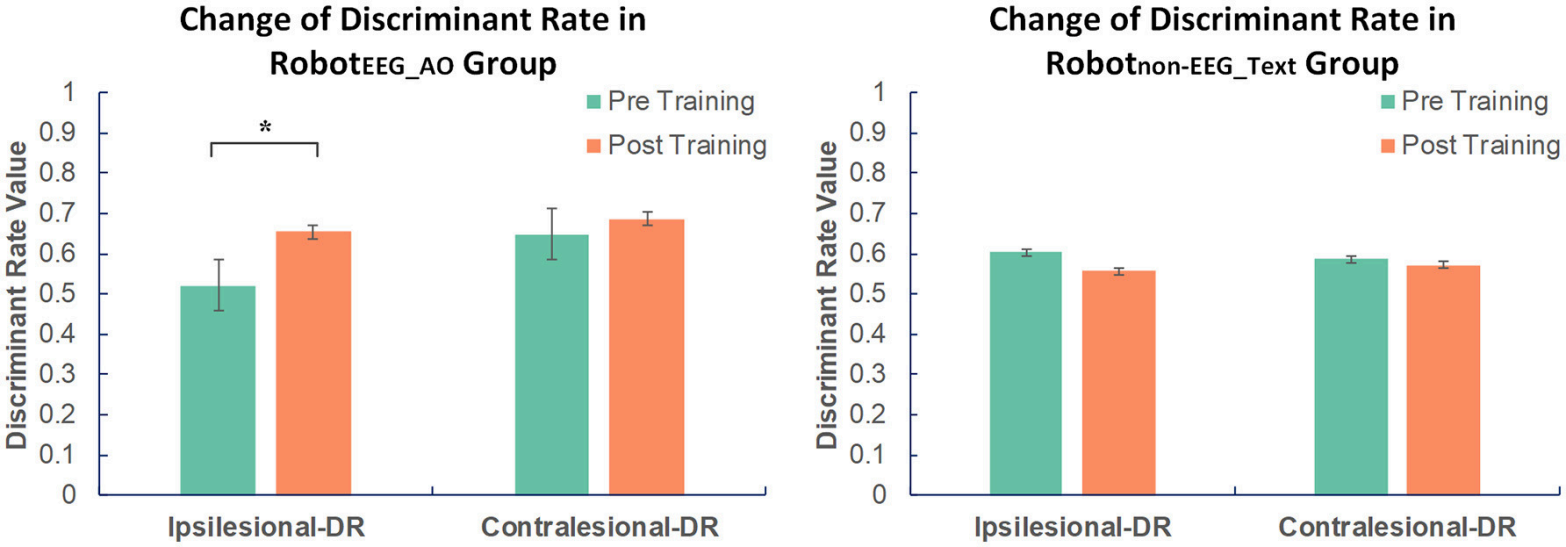
Changes in EEG discriminant rate in two groups before and after intervention. Significant increase in the discriminant rate based on the ipsilesional EEG signals was found after training compared to the baseline in Robot_EEG_AO_ group. No significant change was found in Robot_non−EEG_Text_ group in either the ipsilesional or the contralesional discriminant rate. The color bars indicate the average discriminant rate values across the subjects in each group. DR stands for discriminant rate. Error bars stand for the standard deviation. Asterisk (*) indicates that significant difference between pre- and post-training was observed at *p* < 0.05.

For Robot_EEG_AO_ group, the results of paired *t*-test showed significant increase in the ipsilesional-DR (*t* = 2.762; *p* = 0.018) after the training. However, there was no significant change in the contralesional-DR before and after the training (*t* = 0.757; *p* = 0.465). For Robot_non−EEG_Text_ group, no significant change was found in either the ipsilesional-DR (*t* = −0.765; *p* = 0.462) or the contralesional-DR (*t* = −0.169; *p* = 0.869). The average ipsilesional and contralesional discriminant rates in the two groups before and after training are presented in Figure 3. This indicated that guided robot hand training could enhance motor imagery performance of the stroke subjects using their ipsilesional EEG signals. On the contrary, the non-guided robot hand training did not promote the ability of discriminating between motor imagery state and resting state.

### Network based temporal variability from resting-state fMRI

**Figure 4:**
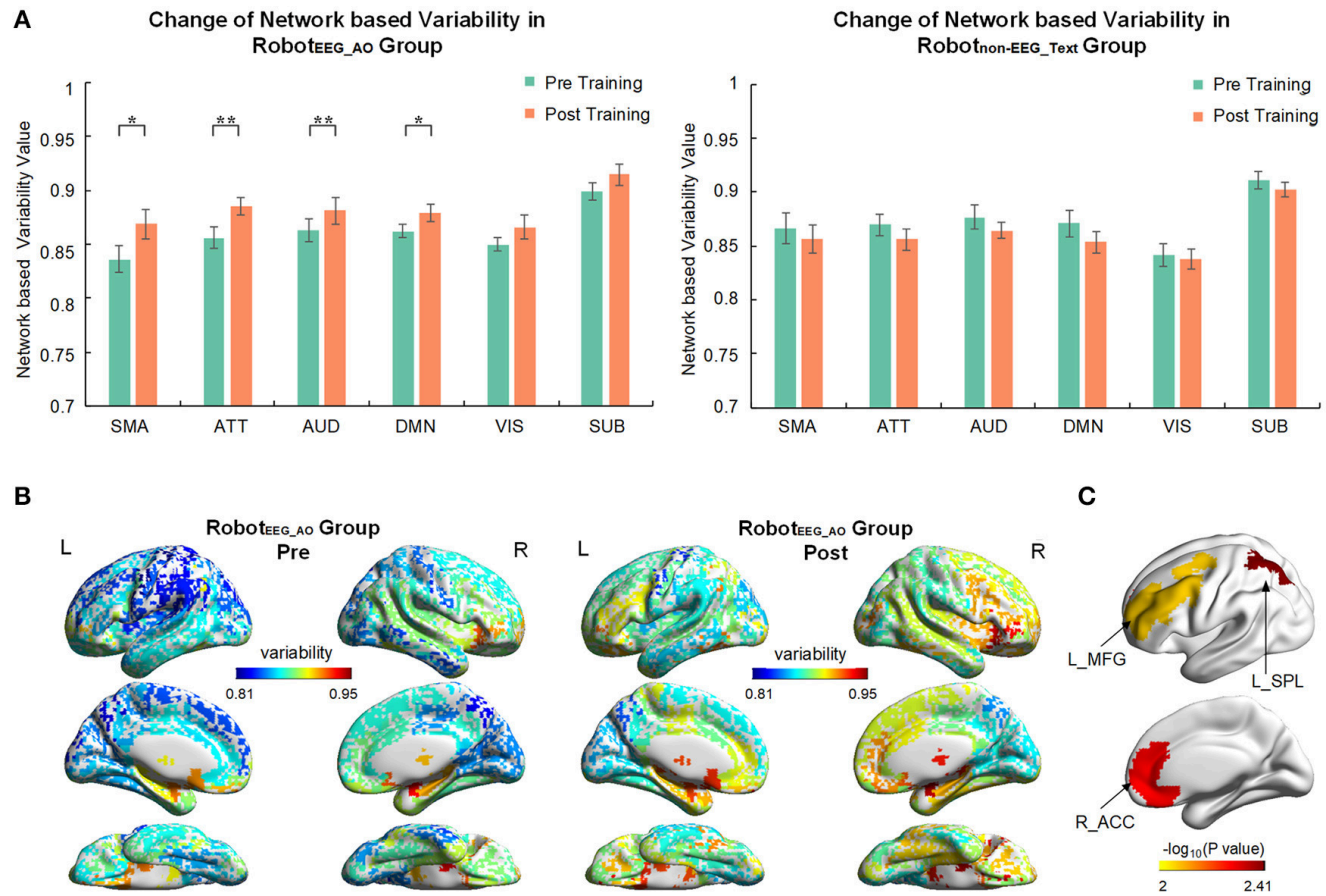
Brain network variability changes after the interventions. **(A)** Comparison of variability in six brain subnetworks between pre and post training in two groups. Only four out of six subnetworks had significant change after training in Robot_EEG_AO_ group while no significant change in any subnetwork in Robot_non−EEG_Text_ group. Error bars are standard errors. *P < 0.05 and **P < 0.01. SMA, sensory-motor areas; ATT, attention network; AUD, auditory network; DMN, default mode network; VIS, visual recognition network; SUB, subcortical network. **(B)** Whole-brain variability topography before and after training for Robot_EEG_AO_ group. Higher variability indicates a more flexible role of one region that may participate in multiple functions. The variability has been averaged across all the subjects in the group. **(C)** Brain regions showing significant increase in variability after the intervention based on the paired *t*-test (*P* < 0.01) for the Robot_EEG_AO_ group. L_MFG, left middle frontal gyrus; L_SPL, left superior parietal lobule; R_ACC, right anterior cingulate cortex.

For the Robot_EEG_AO_ group, the results from time × network repeated measures ANOVA showed significant effect across within-subject time point on the temporal variability of brain regions within the same functional subnetworks, including sensory-motor areas [$F_{\left(1,\;7\right)}=7.554,\;p=0.029,\;\eta^{2}=0.519$], attention network [$F_{\left(1,\;7\right)}=12.354,\;p=0.01,\;\eta^{2}=0.638$], auditory network [$F_{\left(1,7\right)}=13.095,\;p=0.009,\;\eta^{2}=0.652$] and default mode network [$F_{\left(1,\;7\right)}=5.73,\;p=0.048,\;\eta^{2}=0.45$]. No significant effect was found for the visual and subcortical networks. There was also no significant interaction between time and network. For the Robot_non−EEG_Text_ group, no significant main effect was found for either time factor or temporal variability factor of any of the subnetworks. Figure 4A illustrates the brain network variability changes in two groups. For the Robot_EEG_AO_ group, the whole-brain temporal variability topographies before and after the intervention are shown in Figure 4B. Three regions, including right (ipsilesional) anterior cingulate cortex (*p* = 0.0053), left (contralesional) superior parietal lobule (*p* = 0.0039) and left (contralesional) middle frontal gyrus (*p* = 0.009), were found having significant increase in variability after the intervention based on the paired *t*-test (*p* < 0.01), and they are highlighted in Figure 4C. For the between-group comparison, there was no statistically significant difference in temporal variability changes of the six subnetworks between the two groups [*F*_(6, 9)_ = 1.523, *p* = 0.275; Wilk's λ = 0.496, partial η^2^ = 0.504].

## Discussion

This study aimed to compare the longitudinal training effects and neuroplasticity changes in two groups of chronic stroke subjects who received either guided (AO + EEG feedback) or non-guided robot hand training. The changes in brain discriminant ability between the motor imagery state and the resting state revealed from EEG signals, and the changes in brain network variability revealed from resting-state fMRI data were explored as the measures of neuroplasticity changes in the stroke subjects following the two different training strategies. Based on the clinical assessments, there was a statistically significant difference in the paretic upper-limb motor functions across the longitudinal evaluation for the subjects in guided training group, while no significant difference in the long-term training effect was found for the non-guided training group. Higher proportion of stroke subjects exceeding the MCID was also found in the group receiving guided training compared with the non-guided training group. Besides, significant neuroplasticity changes were only observed in the subjects who were trained with guidance. The brain discriminant ability based on the ipsilesional EEG signals significantly improved, and the brain network variability derived from fMRI data also significantly increased in four functional subnetworks, including sensory-motor areas, attention network, auditory network and default mode network after the intervention. These neural measures revealed the differences in the neuroplasticity and brain reorganization in the stroke subjects following the two training strategies. Our findings might imply that the neuroplasticity could be promoted more profoundly by the intervention with neurofeedback, and might be shaped to achieve better motor skill acquisition.

### Motor functional recovery

A significant long-term training effect on the paretic upper-limb motor functional improvement was observed only in Robot_EEG_AO_ group where guided training (AO + EEG feedback) was applied. It should be noted that both the two groups received comparable robot hand training. Previous studies have shown that robot-assisted training alone can promote encouraging upper-limb functional recovery of chronic stroke subjects (1, 54, 55). Therefore, the robot-assisted training without neural guidance could still achieve comparable improvement in paretic upper-limb motor performance at a similar level as the subject group with neural guided training. However, from a long-term perspective, the improvement in the paretic upper-limb motor functions could be more sustainable for the subjects who received guided training compared to those who received non-guided training. Moreover, it is interesting to observe higher proportions of stroke subjects exceeding the MCID in the guided training group. The MCID, which is defined as the smallest change in a treatment outcome that is identified as clinically important to patients or clinicians, is introduced since statistical significance sometimes does not necessarily imply clinical importance. In some cases, retaining a rigid cutoff point (p < 0.05) can induce a drawback that a potential clinically important difference can be denoted as statistically insignificant and ignored due to a small sample size studied (type II errors) (33). The concept of MCID is thus proposed for studying the clinical importance and can be a threshold above which the experienced outcome is treated as relevant to the patient. The results of better motor skill acquisition revealed in the guided training group might be attributed to the synchronization between the peripheral stretching of the paretic hand and the detection of motor imagery with an augmented effects of action observation. It is likely that the centers and motor pathways involved during motor execution could be activated during motor imagery at subthreshold levels (56). The better motor skill acquisition could be associated with the concurrent neuroplasticity changes.

### Motor imagery performance

EEG discriminant rate describes an ability of discriminating brain activity between motor imagery state and resting state, which can also be regarded as an indicator of motor imagery performance in this study. Higher EEG discriminant rate represents a better motor imagery performance and vice versa. Motor imagery performance can be manifested in the classification accuracy of a fixed classification model that can be used to detect the change in motor imagery performance after the intervention. In our design of interventional protocol for EEG guided robot hand training, the subjects were trained to use the correct signals from the ipsilesional motor areas to control the computer system and activate the robot hand. From our results, significant increase in the EEG discriminant rate based on the ipsilesional EEG signals was also found accordingly after the EEG guided training compared to the baseline. The results could follow our expectation, showing that the subjects were trained to have enhanced motor imagery performance using their ipsilesional EEG features as reflected by the significant increase in the ipsilesional discriminant rate. On the other hand, the subjects in non-EEG guided training group were also asked to perform motor imagery during the display of the text instruction. However, no significant change was found in both ipsilesional and contralesional discriminant rates after the training. The results further demonstrate that proper neural guidance to the subjects could be essential and important to guide their motor imagery and induce neuroplasticity in the ipsilesional brain activity.

### Brain network variability

Consistent with the results of EEG discriminant rate, significant increases in the temporal variability were also only found in the guided training group. The temporal variability can be used to track the ongoing dynamics and spontaneous changes of functional connectivity of a region over time. Higher variability of a region means that the region may participate in multiple and diverse functions across the time (27, 28). Disease-specific variability changes have been studied in previous literature (22, 28, 57), indicating that various diseases would induce different patterns of variability compared to the healthy controls. In our study, significant increase in the brain network variability was found in four functional subnetworks, including sensory-motor areas, attention network, auditory network and default-mode network after the intervention.

Functional MRI can offer high spatial resolution data which can allow us to investigate deeper brain regions and neuroplasticity changes compared to EEG signals. Hence, fMRI results could uncover more network changes than the EEG results which the EEG electrodes were only confined to the central motor areas. Moreover, EEG discriminant rate could only show the ipsilesional neuroplasticity changes after intervention, while fMRI results could show increasing trends for both the ipsilesional and contralesional brain regions after the intervention, indicating that the neuroplasticity could occur not only in the ipsilesional hemisphere but also in the contralesional hemisphere in response to guided robot hand training.

The increased variability in sensory-motor areas after guided training is consistent with previous findings showing that increased sensorimotor autonomy was found during skill acquisition, displaying a neural-efficiency state (58). In line with the inference from the results of EEG discriminant rate, the EEG signals from the ipsilesional motor areas were used to activate the robot hand in the interventional protocol design, and therefore it is reasonable that significant variability increase in the sensory-motor areas was also revealed after the intervention.

Apart from the sensory-motor areas, significant variability changes were also revealed in the attention, auditory and default mode networks after the intervention. Action observation was provided during neural guided training, which would activate the mirror neuron system (a fronto-parietal neural circuit) that is active during both action observation and execution (59). The flexibility of the three regions, including anterior cingulate cortex, superior parietal lobule and middle frontal gyrus, was strengthen after the intervention (Figure 4C). These regions, which had significant variability changes after the intervention, have been documented to contain mirror neurons, constituting the human mirror neuron system. The impaired motor system after stroke may become accessible by recruiting the shared motor circuits during action observation. Repetitive mental practice might enhance these regions for their re-adaptation to participate in multiple functions, possibly contributing to the increase in their temporal variability. The results could again follow our expectation, suggesting that the mirror neuron system could be enhanced and motor relearning could be achieved by our intervention. Moreover, previous studies have also observed the involvement of parietofrontal areas during stroke recovery (60, 61), which might support the increased variability of these regions in association with the motor functional recovery after the intervention. These regions might play a crucial role in attentional processes related to self-monitored movement (62). Therefore, the dynamic characteristics of these subnetworks could be modulated by the intervention, which is in line with the EEG results showing significant improved discriminant ability between motor imagery and resting states.

## Limitations

Motor recovery from chronic stroke is often difficult and challenging to be achieved and maintained. This study showed that a significant long-term training effect on the paretic upper-limb motor functions could be observed for the subjects who received the guided robot hand training. Moreover, higher proportions of stroke subjects exceeding the clinical significant improvement by using MCID was found in the group receiving guided training compared with the non-guided training group. However, there is also a wide range of available methods for determining the MCID, which would create a major problem that a number of MCID scores are available for a single outcome measure. This would also create difficulty in the interpretation when deciding which MCID score is the most appropriate (63). Despite the fact that the MCID can be varied according to different definition methods, it is independent of treatment methods and therefore two different treatments can be compared using the same MCID for the same outcome measure. Besides, this pilot study examined a total of 24 chronic stroke subjects of which only 16 subjects had the MRI examination. This small sample size would limit the generalization of the findings to a larger population, and the dynamic functional connectivity changes revealed from the 16 subjects who had the MRI examination might also not be able to reflect the whole picture of neuroplasticity changes in the all 24 subjects. The heterogeneous subject demographics, including the variations in lesion site and size, time since stroke and stroke type, may also contribute to the differences in the electrophysiological and the dynamic functional connectivity patterns. In spite of this, both our EEG and fMRI results generally revealed the significant neuroplasticity changes only in the subjects who received the guided training. Nevertheless, future studies with larger sample size will be needed to validate and extend the preliminary findings of this study. Moreover, the study design was a bit ambiguous with the involvement of several influencing factors in the training modes, including the motor imagery in combination with action observation or with text cue, and the EEG-based or random triggering mode of the robot hand. The study outcomes or results could be clearer if each factor was studied separately with more separate subject groups.

## Conclusion

This pilot study uncovered the differences in longitudinal training effect and neuroplasticity changes under two kinds of training strategies for stroke rehabilitation by investigating the changes in the dynamic temporal characteristics of brain regions using resting state fMRI and the brain discriminant ability between motor imagery state and resting state using EEG. Our findings indicated that a sustainable motor functional improvement could be achieved through proper guidance and neurofeedback to the stroke subjects. Moreover, neural guidance could be essential and important to induce neuroplasticity, and the neuroplasticity could occur in multiple brain networks involving both the ipsilesional and contralesional hemispheres.

## Author contributions

XW and RS performed experiments, analyzed data, and wrote the paper. WW designed, performed experiments, analyzed data, and wrote the paper. WC and K-YT designed experiments. All authors reviewed the manuscript.

### Conflict of interest statement

The authors declare that the research was conducted in the absence of any commercial or financial relationships that could be construed as a potential conflict of interest.
