# Supplementary material for: Differentiated Effects of Robot Hand Training With and Without Neural Guidance on Neuroplasticity Patterns in Chronic Stroke
Source: Front Neurol. 2018 Oct 8;9:810. doi: 10.3389/fneur.2018.00810 (PMC6186842; doi:10.3389/fneur.2018.00810)
Supplement: Supplementary file 1 [file Table_1.pdf]

*Supplementary Material*

# Differentiated Effects of Robot Hand Training With and Without Neural Guidance on Neuroplasticity Patterns in Chronic Stroke

**Xin Wang[1], Wan-wa Wong[1], Rui Sun[1], Winnie Chiu-wing Chu[2], Raymond Kai-yu Tong[1,3,*]**

*** Correspondence:** Raymond Kai-yu Tong: kytong@cuhk.edu.hk

## 1 Supplementary Table

**Supplementary Table 1** Region of Interest with corresponding subnetworks

| Index | FreeSurfer Labels | AAL Labels | Subnetworks |
|---|---|---|---|
| 1 | Superior frontal gyrus (F1) | Frontal Sup<br>Frontal Sup Med | Default Mode Network |
| 2 | Anterior part of the cingulate gyrus and sulcus (ACC) | Frontal Med Orb<br>Cingulum Ant | |
| 3 | Straight gyrus<br>Gyrus rectus | Rectus | |
| 4 | Posterior-dorsal part of the cingulate gyrus (dPCC) | Cingulum Post | |
| 5 | Angular gyrus | Angular | |
| 6 | Precuneus | Precuneus | |
| 7 | Middle temporal gyrus (T2) | Temporal Mid | |
| 8 | Temporal pole | Temporal Pol Mid | |
| 9 | Precentral gyrus | Precentral | Sensory-Motor Areas |
| 10 | Postcentral gyrus | Postcentral | |
| 11 | Paracentral lobule and sulcus | Paracentral Lob | |
| 12 | Orbital gyri | Frontal Sup Orb<br>Frontal Inf Orb | Attention Network |
| 13 | Middle frontal gyrus (F2) | Frontal Mid | |
| 14 | Fronto-marginal gyrus (of Wernicke) and sulcus | Frontal Mid Orb | |

| | | | |
|---|---|---|---|
| 15 | Opercular part of the inferior frontal gyrus | Frontal Inf Oper | |
| 16 | Triangular part of the inferior frontal gyrus | Frontal Inf Tri | |
| 17 | Superior parietal lobule (lateral part of P1) | Parietal Sup | |
| 18 | Intraparietal sulcus (interparietal sulcus) and transverse parietal sulci | Parietal Inf | |
| 19 | Subcentral gyrus (central operculum) and sulci | Rolandic Oper | Auditory Network |
| 20 | Superior segment of the circular sulcus of the insula | Insula | |
| 21 | Short insular gyri | Insula | |
| 22 | Supramarginal gyrus | SupraMarginal | |
| 23 | Anterior transverse temporal gyrus (of Heschl) | Heschl | |
| 24 | Lateral aspect of the superior temporal gyrus | Temporal Sup | |
| 25 | Planum polare of the superior temporal gyrus | Temporal Pole Sup | |
| 26 | Calcarine sulcus | Calcarine | Visual Network |
| 27 | Cuneus (O6) | Cuneus | |
| 28 | Lingual gyrus, ligual part of the medial occipito-temporal gyrus, (O5) | Lingual | |
| 29 | Superior occipital gyrus (O1) | Occipital Sup | |
| 30 | Middle occipital gyrus (O2, lateral occipital gyrus) | Occipital Mid | |
| 31 | Inferior occipital gyrus (O3) and sulcus | Occipital Inf | |

| | | | |
|---|---|---|---|
| 32 | Lateral occipito-temporal gyrus (fusiform gyrus, O4-T4) | Fusiform | |
| 33 | Subcallosal area, subcallosal gyrus | Olfactory | Subcortical Network |
| 34 | Middle-posterior part of the cingulate gyrus and sulcus (pMCC) | Cingulum Mid | |
| 35 | Hippocampus,Amygdala | Hippocampus | |
| 36 | Parahippocampal gyrus, parahippocampal part of the medial occipito-temporal gyrus, (T5) | ParaHippocampal | |
| 37 | Amygdala | Amygdala | |
| 38 | Caudate | Caudate | |
| 39 | Putamen | Putamen | |
| 40 | Pallidum | Pallidum | |
| 41 | Thalamus-Proper | Thalamus | |
| 42 | Inferior temporal gyrus (T3) | Temporal Inf | |
