# Supplementary material for: Differentiated Effects of Robot Hand Training With and Without Neural Guidance on Neuroplasticity Patterns in Chronic Stroke
Source: Front Neurol. 2018 Oct 8;9:810. doi: 10.3389/fneur.2018.00810 (PMC6186842; doi:10.3389/fneur.2018.00810)
Supplement: Supplementary file 2 [file Image_1.pdf]

Supplementary Material

Differentiated Effects of Robot Hand Training With and Without Neural Guidance on Neuroplasticity Patterns in Chronic Stroke

Xin Wang[1], Wan-wa Wong[1], Rui Sun[1], Winnie Chiu-wing Chu[2], Raymond Kai-yu Tong[1,3,*]

*** Correspondence:** Raymond Kai-yu Tong: kytong@cuhk.edu.hk

1 Supplementary Figures

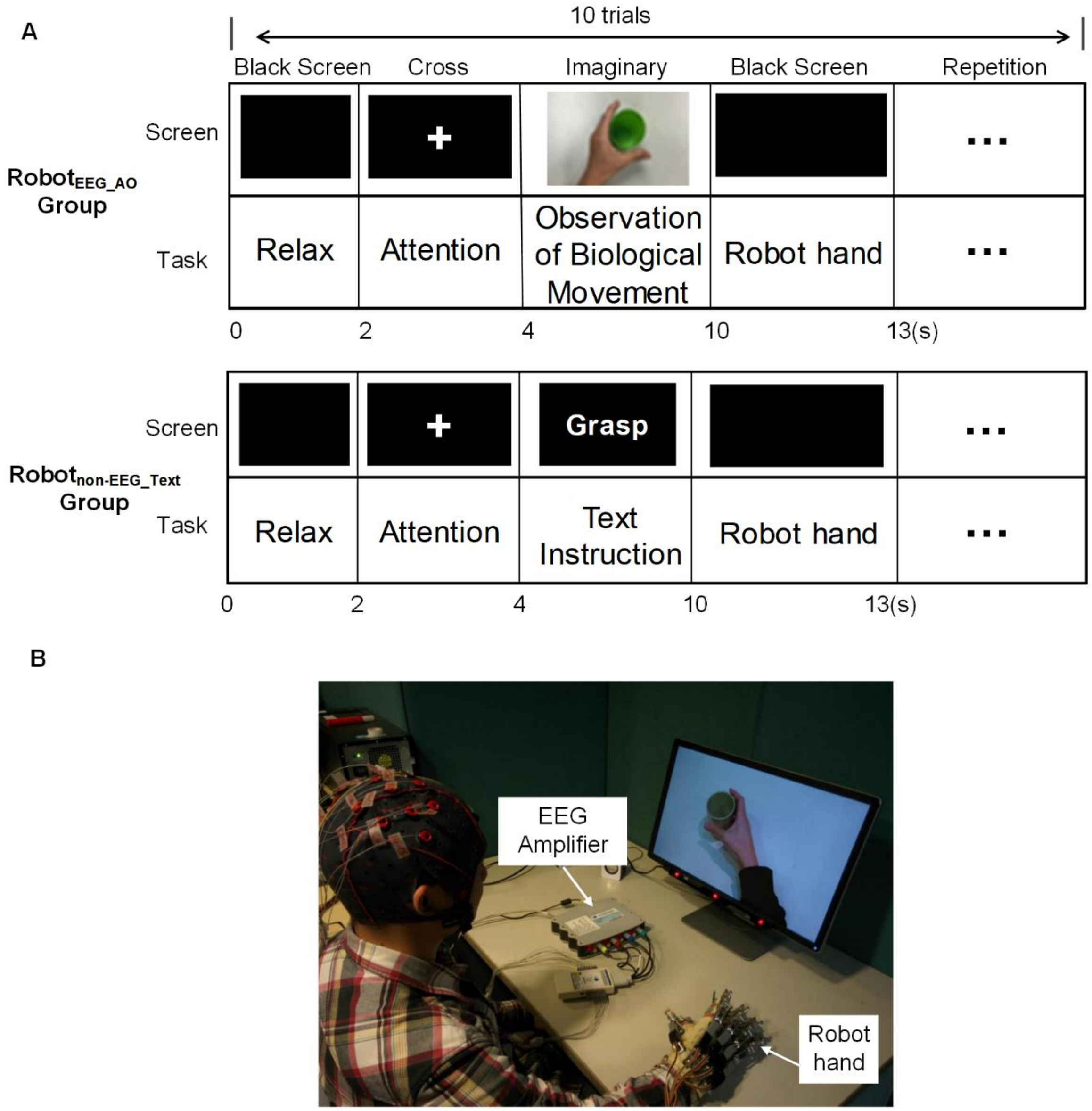

Supplementary Figure 1. Experimental setup. **(A)** Experimental paradigm for two groups. During each session, 100 repetitive movements were performed by each subject with intermittent rest after every 10 trials. For Robot$_{EEG_AO}$ group, real-time EEG were collected during imaginary and used to guide and trigger the robot hand to help grasp or release. During their imaginary, a video demonstrating either grasping or releasing a cup using the subjects' unaffected hand was applied as action observation. For Robot$_{non\text{-}EEG_Text}$ group, no guidance from the EEG and the robot hand was triggered randomly. During their imaginary, only text instruction rather than action observation was applied. **(B)** The training system for hand training.
