# Supplementary material for: Differentiated Effects of Robot Hand Training With and Without Neural Guidance on Neuroplasticity Patterns in Chronic Stroke
Source: Front Neurol. 2018 Oct 8;9:810. doi: 10.3389/fneur.2018.00810 (PMC6186842; doi:10.3389/fneur.2018.00810)
Supplement: Supplementary file 3 [file Image_2.pdf]

Supplementary Material

Differentiated Effects of Robot Hand Training With and Without Neural Guidance on Neuroplasticity Patterns in Chronic Stroke

Xin Wang[1], Wan-wa Wong[1], Rui Sun[1], Winnie Chiu-wing Chu[2], Raymond Kai-yu Tong[1,3,*]

*** Correspondence:** Raymond Kai-yu Tong: kytong@cuhk.edu.hk

1 Supplementary Figures

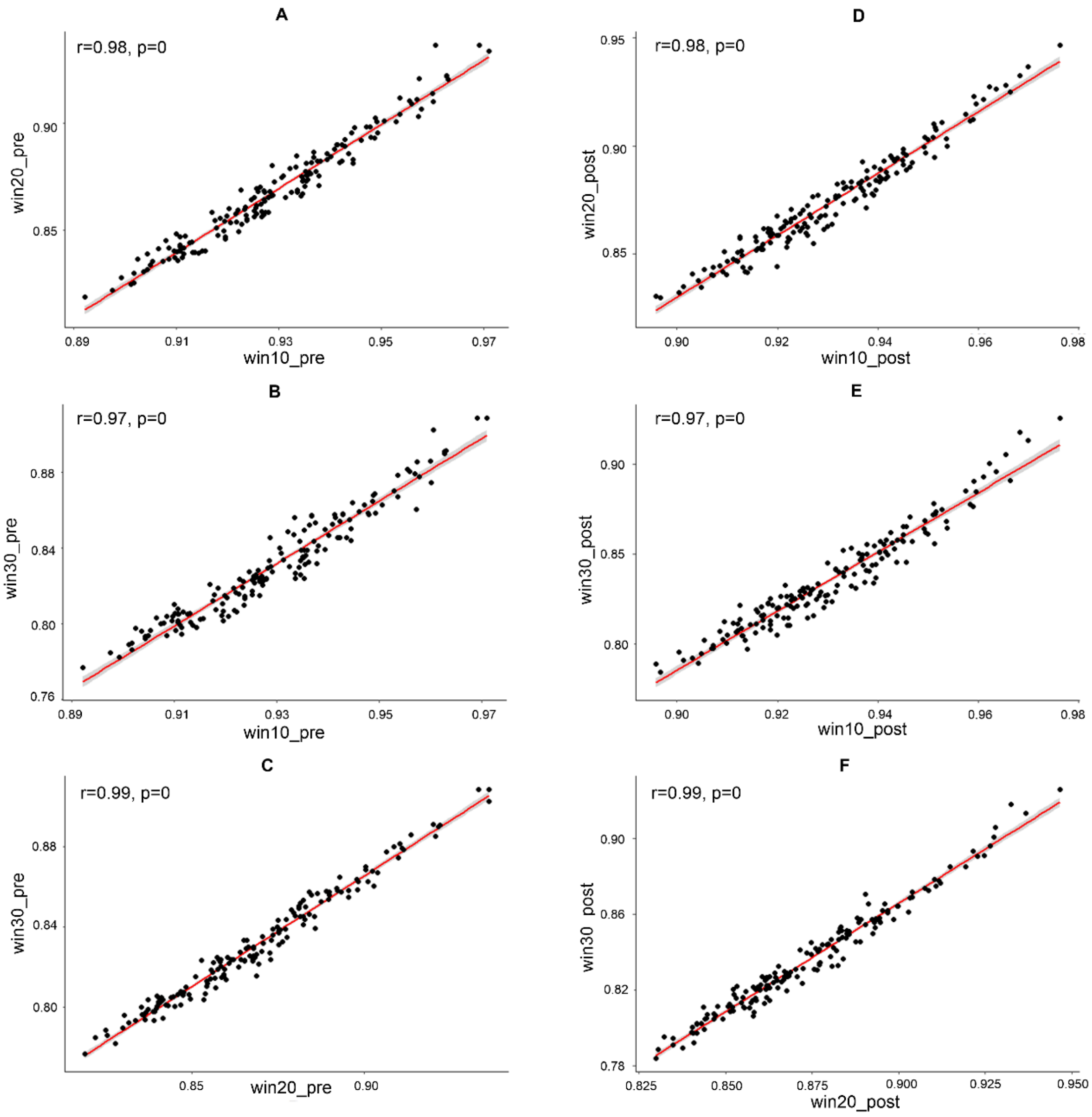

Supplementary Figure 2. Scatter plot of variability correlations between different window length (10, 20, and 30 time points) before intervention (A, B and C) and after intervention (D, E and F). Each point in the figure represents a region, and the variability is averaged among subjects for group 1. “win10_pre” and “win10_post” denotes variability obtained using time window length being 10 time points in that window before intervention and after intervention respectively. High correlation relationships can be found among different window length from the plot. Thus, it is reasonable to average the variability in this range of window length.
